# Fracture risk in dialysis and kidney transplanted patients: a protocol for systematic review and meta-analysis

**DOI:** 10.1186/s13643-017-0416-8

**Published:** 2017-02-22

**Authors:** Aboubacar Sidibé, Lynne Moore, Sonia Jean, Fabrice Mac-Way

**Affiliations:** 1 Centre de Recherche du CHU de Québec, Hôpital Hôtel-Dieu de Québec, Division of Nephrology, Endocrinology and Nephrology Axis, Faculty and Department of Medicine, Laval University, 10 McMahon, Québec City, Québec G1R 2J6 Canada; 2 Centre de Recherche du CHU de Québec, Hôpital de l’Enfant-Jésus, Traumatology Axis, Medicine Faculty, Department of Social and Preventive Medicine, Laval University, Quebec, Canada; 3 Institut National de Santé Publique du Québec, Medicine Faculty, Department of Social and Preventive Medicine, Laval University, Quebec, Canada

**Keywords:** Fractures, Hemodialysis, Peritoneal dialysis, Kidney transplantation

## Abstract

**Background:**

Chronic kidney disease (CKD) is associated with an increased risk of fracture and cardiovascular mortality. The risk of fracture in hemodialysis (HD), peritoneal dialysis (PD), and kidney transplantation (KT) is higher when compared to the general population. However, uncertainties remain about which group has the highest risk of fracture. We aim to identify the risk of fracture and cardiovascular mortality post-fracture in HD compared to PD or KT and in PD compared to KT population.

**Methods:**

We will conduct a systematic review of observational studies and randomized control trials on patients with CKD. Eligible studies will be searched on MEDLINE, Embase, Web of Science, Cochrane Library, and in gray literature. Two independent reviewers will screen all identified references in order to include studies reporting the risk of fracture without a comparator or comparing that risk in HD vs KT, PD vs KT, or HD vs PD. Studies comparing the risk of fracture in a renal replacement therapy group to general population or to non-dialyzed CKD patients will also be included. Data on study settings, population characteristics, intervention, comparator, and outcomes will be extracted. Study data will be summarized and analyzed in RevMan and SAS. Risk of bias in cohort design studies will be assessed with an adapted version of the ROBINS-I tool and by the Cochrane handbook tool for RCTs. The quality of evidence and strengths of recommendations will be evaluated by the Grading of Recommendations Assessment, Development and Evaluations (GRADE) tool. We will pool relative risks with random-effect models and Mantel-Haenszel methods. Subgroup and sensitive analysis are planned according to the intervention and comparator, study design, and type of fracture.

**Discussion:**

This review will provide new pooled data about fracture risk in dialysis and KT patients. Our results should guide the implementation of future preventive strategies targeting patients with the highest fracture risk. A pooled analysis of observational studies could be limited by a probable considerable heterogeneity among these studies.

**Systematic review registration:**

PROSPERO CRD42016037526

**Electronic supplementary material:**

The online version of this article (doi:10.1186/s13643-017-0416-8) contains supplementary material, which is available to authorized users.

## Background

Chronic kidney disease (CKD) is becoming a major public health issue worldwide. In 2011, more than 615,000 people suffered from end-stage renal disease (ESRD) in the USA [1]. In 2013, statistics in Canada estimated that 41,914 Canadians suffered from ESRD [2]. Kidney function decline leads to metabolic disorders that affect bone metabolism and vascular health known as CKD-mineral and bone disorder (CKD-MBD) [3]. Clinically, CKD-MBD has been associated with an increased risk of fracture and cardiovascular mortality post-fracture [4–8].

Patients with ESRD will eventually require renal replacement therapy [9]. Therefore, these patients are treated by hemodialysis (HD), peritoneal dialysis (PD), or kidney transplantation (KT). In 2013, the proportion of patients treated by each modality was 47.37 vs 10.13% vs 42.51%, respectively, in Canada [2]. The increased risk of fracture in HD, PD, and KT patients, compared to the general population is well documented [4, 10, 11]. Indeed, the risk of hip fracture in ESRD is estimated to be 4- to 14-fold the risk seen in the general population [4, 11]. However, there exists a knowledge gap on the risk of fracture between these three groups of patients.

While Beaubrun et al. [12] reported in the USA that the incidence rate of hip fractures in HD patients was 20.6 per 1000 person-years in 2009, Nair et al. [13], in the same design study, reported a much lower incidence rate in KT patients, estimated at 3.8 per 1000 person-years. In contrast, another study has reported that the risk of hip fracture was in fact 1.34-fold higher in KT vs dialysis patients [14]. When comparing patients in dialysis, a recent study [15] has found that the risk of hip fracture in HD was 1.74-fold higher than that in PD, while another study did not find any difference between HD, PD, and KT patients [5]. Given these finding disparities, a systematic review is required to provide clear information on the incidence of fracture in dialysis and kidney transplant population.

## Why is it important to do this review?

Uncertainties remain concerning the risk of fracture between HD, PD, and KT patients. It is clinically important to obtain these data in order to implement fracture prevention strategies and treatments in this population. Based on previous available literature, one of the reasons that could explain the higher risk of fractures in HD patients might be their increased number of comorbidities as compared to PD and KT population. On the other hand, KT patients may also have a higher fracture risk due to the use of glucocorticoids to reduce the risk of graft rejection [16, 17]. Clearly, this review will provide new, robust, and clinically useful data on the incidence of fracture in HD, PD, and KT patients and whether the risk of fracture is different.

## Objectives

### Primary objective

Identify the risk of fracture in HD compared to PD or KT and in PD compared to KT populations.

### Secondary objective

Identify the risk of cardiovascular mortality post-fracture in HD compared to PD or KT and in PD compared to KT populations.

## Methods

We will conduct a systematic review based on the preferred reporting items for systematic reviews and meta-analyses (PRISMA) statement [18] and the Cochrane handbook for systematic reviews of interventions methodological recommendations [19]. This protocol is based on the checklist of the PRISMA-P for systematic reviews protocol [20] (See Additional file 1).

## Eligibility criteria

### Study design

We will include in our review randomized control trials (RCTs) (even if they are not likely to provide us with the data we seek) and observational studies (cohort studies, cross sectional studies, case-control studies). Meeting abstracts will be included in our review only if they provide all the necessary information. Case report studies will be excluded.

### Type of participants

To be included in our review, studies should have included adults ≥18 years (at least 80% of participants) of CKD treated by either hemodialysis, kidney transplantation, or peritoneal dialysis.

### Type of intervention

The intervention of interest is renal replacement therapy (HD, PD, and KT).

### Comparator

To be included in our review, the comparator used should be either no comparator group, general population, non-dialyzed CKD, or a renal replacement therapy (HD, PD, KT).

### Type of outcome

#### Primary outcome

The primary outcome will be the risk (incidence rate, incidence proportion, odds, or prevalence) of fracture (hip, vertebral, and any fracture).

#### Secondary outcomes

1. Fracture sites (hip, vertebral, non-vertebral).
2. Risk of cardiovascular mortality post-fracture.
3. All-cause mortality associated with fracture.
4. Length of hospitalization post-fracture.
5. Number of hospitalizations post-fracture (during the following year).

## Information sources

We will systematically search MEDLINE, Embase, Web of science, and The Cochrane Library (from their inception up to a maximum of 6 months’ prior submission for publication, in order to include as many trials as possible) for studies eligible to our review. We will consult Google Scholar and thesis repositories to identify additional studies, including Thesis Canada Portal, EtHOS, DART-Europe E-Theses Portal, the National Library of Australia’s TROVE, and ProQuest Dissertations & Theses Global.

## Search strategy

The search strategy is based on keywords related to the intervention (hemodialysis, peritoneal dialysis, kidney transplantation, and all synonyms) and related to the outcome (keywords related to fracture and synonyms). A strategy has been first established for PubMed and Embase and adapted to other databases. We will not set any restrictions for the language or year of publication. An additional file shows the search strategy established for PubMed in more details (see Additional file 2).

## Data management and selection of studies

Data will be imported using EndNote software (version X7.2.1, New York, NY, Thomson Reuters, 1988–2014), where duplicates will be removed. Unique references will then be exported in Excel software (Excel software 2016, Microsoft office 2016). An additional file is provided to describe the procedure of exportation from EndNote to Excel (see Additional file 3). Two review authors will independently screen each study by title, abstract, and full text if necessary for inclusion in the review using the inclusion criteria described above. Disagreements will be resolved by consensus between reviewers or with the involvement of a third reviewer. Studies not written in French or English will be translated when full text review will be needed, either by review collaborators or the linguistic department of Laval University.

## Data collection process

Data extraction will be performed using an abstraction form created in Excel (Excel software 2016, Microsoft office 2016). A preliminary version of that form will be pilot-tested and customized by two reviewers using five publications, and the standardized form will be used for full abstraction. In case of discrepancy, consensus will be reached with the involvement of a third reviewer.

## Incomplete data

We plan to contact the investigators in case of missing information or if explanation about an important variable is needed. If information on a secondary outcome variable cannot be obtained, the study will not be included in analyses of that outcome. If an included study reports incomplete information (e.g., means available but no standard deviations), we will attempt to recalculate values using available data on RevMan software or with statistical approach proposed by Hozo et al. [21].

## Data items

We will extract information on the study (name of the first author and co-authors, study design, publication year, publication journal, language of publication, and source of funding), characteristics of the study population (sample size, age, sex, stage of CKD, comorbidities, duration of illness, past history of fracture, type of drugs used, treatment center (hemodialysis center, kidney transplant unit, nephrology department, home dialysis center), intervention (HD, PD, KT, duration of dialysis, duration of dialysis before KT, time spent on KT waiting list), type of comparator (HD, PD, KT, general population, non-dialyzed CKD), and outcomes (number of fractures, diagnosis method for fracture, risk of fracture, the risk of cardiovascular and overall mortality post-fracture, length of hospitalization post-fracture, number of hospitalizations and complications post-fracture, fracture sites and types).

## Assessment of risk of bias in included studies

Methodological quality (internal validity) of RCTs will be assessed using the Cochrane Collaboration Risk of Bias Tool [22]. Risk of bias in observational studies will be assessed with ROBINS-I tool [23] adapted to our review by the steering committee (comprising two clinicians, an epidemiologist, a biostatistician, and a review specialist). This tool consists of six domains (bias due to confounding, bias in selection of participants into study, bias in classification of interventions, bias due to missing data, bias in measurement of outcomes, bias in selection of the reported results) and the interpretation of domain-level and overall risk of bias judgements. Each separate domain is rated as “low”, “moderate”, “serious”, “critical”, or “no information”. The overall assessment is based on the responses to individual domains.

## Summary measures

We will report crude and adjusted risk (prevalence, incidence, rate incidence) of fracture and cardiovascular mortality post fracture according to intervention and comparators. For risk of fracture, we considered adjustment for age, sex, and antecedent of fracture (if applicable) to represent optimal control for confounders and any reported adjustment to represent minimal control for confounders. Continuous variables such as age, mean duration of CKD, duration of follow-up will be reported using means and standard deviations.

## Data synthesis

The study data will be summarized and analyzed with Review Manager Software (Revman, Computer program, Version 5.3 Copenhagen: The Nordic Cochrane Centre, The Cochrane Collaboration, 2014). Data from similar studies (using similar measures of frequencies and comparing the same outcome in the same groups) will be combined in meta-analysis if at least three studies are available by outcome of interest. However, we expect that studies will use different measure of frequencies for the outcomes according to the study design. We also expect that the duration of follow-up will be different between studies. Consequently, cumulative incidence or odds (for risk <0.10) will be converted to incidence rates using the statistical approach recommended by Rothman [24] and pooled together. This approach will account for differences in follow-up duration between studies. Studies reporting the prevalence of fracture will be pooled separately. For continuous data, we will use inverse variance method with random effect models to pool the standardized mean difference if studies used different scales for the assessment of the same outcome or mean difference if studies used the same scale. For dichotomous variables, the data from individual studies will be combined using Mantel-Haenszel method with random effects models to pool relative risks). Pooled effect sizes and their 95% confidence limits will be reported. If quantitative synthesis is not appropriate, studies will be described individually according to intervention, comparator, and outcomes reported in summary tables.

## Measures of treatment effect

For dichotomous variables, the treatment effect will be calculated using a Mantel-Haenszel random effects model to estimate pooled relative risks. For continuous data, we will calculate the treatment effect using random effects of inverse variance to estimate the standardized mean difference where studies used different scales for the assessment of the same outcome and mean difference where studies used the same method to measure the outcome. All estimates will be presented with 95% confidence intervals.

## Unit of analysis issues

The overall risk of fracture, risk for specific sites of fracture, cardiovascular and all-cause mortality post-fracture, and incidence of hospitalization post-fracture will be analyzed as dichotomous variables. Length of hospital stay post-fracture will be analyzed as continuous variables.

## Additional analysis

Statistical heterogeneity will be assessed with the *Q* test and Cochrane’s I^2^ [25]. Heterogeneity will be interpreted as low between 0–30%, moderate between 30–60%, considerable >60% [19].

Clinical outcomes will be analyzed in subgroups according to the intervention and comparison (HD vs KT, HD vs PD, PD vs KT), study design, and type or site of fracture.

## Sensitivity analysis

We will carry out sensitivity analyses to explore the influence of publication type on results. A sensitivity analysis will also be performed by removing studies that have high or unclear risk of bias for RCTs studies and by removing studies with a serious or critical risk of bias for observational studies in order to evaluate the robustness of results to potential bias assessed with the ROBINS-I tool.

## Meta-regression

In case of a considerable heterogeneity among studies, a meta-regression will be performed if the number of studies is sufficient (>10 by covariate) [19]. Covariates of interest will be duration of dialysis or kidney transplantation, age of participants, percentage of women, type of treatments used in dialysis, and after kidney transplantation (corticosteroids, bisphosphonate, vitamin D, phosphate chelators), comorbidities (diabetes, hypertension, obesity, smoking status), cause of ESRD, and ethnicity. These analyses will be performed using PROC MIXED (SAS 9.4) [26].

## Meta-bias

We will attempt to avoid reporting bias by using a sensitive and reproducible search strategy, including as many keywords and synonyms as possible. We will also assess the risk of publication bias with funnel plots if at least 10 studies comparing the same groups of treatment are included as recommended by the Cochrane handbook [27]. We will also evaluate the risk of selective reporting of outcomes within studies by searching for previously published protocols on registration website (www.controlled-trials.com and clinicaltrials.gov).

## Quality of evidence

Overall quality of evidence will be assessed using the Grading of Recommendations, Assessment, Development and Evaluations (GRADE) tool as recommended by the Cochrane handbook for systematic reviews of intervention [19].

## Discussion

### Expected benefits

Bone disease in CKD is a major health issue leading clinically to increased fracture rates and higher societal costs due to prolonged length of hospitalization and number of hospitalization post-fractures [12]. This review will provide new pooled data on the fracture risk between three high-risk groups of CKD patients that are on renal replacement therapy. It will allow us to identify the group most associated with fracture (if there is a difference) while the results will potentially reinforce the importance of fractures in CKD population.

### Inform future studies

Considering heterogeneity in methodologies used in observational studies and the risk of bias associated with these studies, we expect the results to be heterogeneous. Our results will therefore guide the implementation of future preventive strategies targeting patients with high fracture risk in CKD. Moreover, it will enable future research to focus on new therapeutic treatments aiming at reducing fracture risk in CKD population. Finally, the provided details about the methodology used in included studies in this review will help future studies in improving their methodology quality.

### Limitations

Some elements could represent limitations for the pooled analysis of this systematic review such as (1) the statistical and methodological heterogeneity between observational studies, (2) the type of fracture and methods used for fracture’s diagnosis, and (3) we may obtain unadjusted or incompletely adjusted effect estimates. Also, the expected number of studies at low risk of bias could be low. The strength of evidence of our results may thus be limited by these factors. If sufficient data are available, we will conduct meta-regression analysis, which will help to, at least partially, control for potential confounding bias. Finally, our secondary objective may be limited by the fact that we are likely to miss an important number of studies conceived specifically to estimate the association between the type of renal replacement therapy and the risk of post-fracture cardiovascular or overall mortality.

## Additional files

Additional file 1: PRISMA-P (preferred reporting items for systematic review and meta-analysis, checklist of items that should be found in the manuscript. (PDF 260 kb)
Additional file 2: Search strategy on PubMed, description of the combination of the keywords and synonyms used in PubMed. (PDF 146 kb)
Additional file 3: Procedure of exportation from EndNote to Excel, description of how data is exported from EndNote software to an Excel file. (PDF 135 kb)

## Abbreviations

CKD: Chronic kidney disease
CKD-MBD: Chronic kidney disease-mineral and bone disorder
ESRD: End-stage renal disease
HD: Hemodialysis
KT: Kidney transplantation
PD: Peritoneal dialysis
PRISMA: Preferred reporting items for systematic reviews and meta-analyses
RCTs: Randomized control trials
