# Supplementary material for: Fracture risk in dialysis and kidney transplanted patients: a protocol for systematic review and meta-analysis
Source: Syst Rev. 2017 Feb 22;6:37. doi: 10.1186/s13643-017-0416-8 (PMC5320734; doi:10.1186/s13643-017-0416-8)
Supplement: Additional file 2: — Search strategy on PubMed, description of the combination of the keywords and synonyms used in PubMed. (PDF 146 kb) [file 13643_2017_416_MOESM2_ESM.pdf]

<u>Search strategy for Pubmed</u>

Intervention

1. “Renal Replacement Therapy” [mesh] OR
   ((“Renal replacement”[TIAB] or “Kidney replacement”[tiab]) and (“therapy”[tiab] or “therapies”[TIAB])) OR

   ((“Kidney”[TIAB] or “Renal”[TIAB]) and (transplantation*[TIAB] or “dialysis”[TIAB] or “dialyses”[TIAB] or graft*[TIAB])) OR

   “hemodialysis”[TIAB] OR

   “hemodialyses” [TIAB] OR

   “Peritoneal Dialysis”[TIAB] OR

   “Peritoneal Dialyses”[TIAB] OR

   “Extracorporeal Dialyses”[tiab] OR

   “Extracorporeal Dialysis”[tiab] OR

   “hemodiafiltration”[TIAB]

Outcome

2. “Fractures, Bone”[mesh] OR

((“Broken”[tiab] or fracture*[tiab]) AND (Bone*[tiab] OR “Ankle”[tiab] OR “Malleolus”[tiab] or “Trimalleolar”[tiab] or “Bimalleolar”[tiab] or “Femoral”[tiab] or “Hip”[tiab] or “Subtrochanteric” [tiab] or “Trochanteric”[tiab] or “Intertrochanteric”[tiab] or “Femur Neck”[tiab] or “Spontaneous”[tiab] or Pathologic*[tiab] or “Humeral”[tiab] or “Intra Articular” [tiab] or “Intraarticular”[tiab] or “Radius”[tiab] or “Rib”[tiab] or “Shoulder”[tiab] or “Spinal”[tiab] or “vertebral”[tiab] or “Tibial”[tiab] or “Ulna”[tiab]))

3. #1 AND #2
4. “Animal”[mesh] not “human”[mesh]
5. #3 NOT #4
