# Supplementary material for: Fracture risk in dialysis and kidney transplanted patients: a protocol for systematic review and meta-analysis
Source: Syst Rev. 2017 Feb 22;6:37. doi: 10.1186/s13643-017-0416-8 (PMC5320734; doi:10.1186/s13643-017-0416-8)
Supplement: Additional file 3: — Procedure of exportation from EndNote to Excel, description of how data is exported from EndNote software to an Excel file. (PDF 135 kb) [file 13643_2017_416_MOESM3_ESM.pdf]

<u>Procedure of data exportation from Endnote to Excel</u>

References will be exported from Endnote to Excel by creating a new reference style. We will first create a new references style. From **«Edit»** we will choose **Output Styles** and edit annotated. We will then click on **Templates** among the choices under **«Bibliography»** and choose **«Journal article»** or other type of article according to those among our references. After that, we will change the options for our journal type such as **«Journal Article** » to obtain a profile with an arrow. We will add new tabs by clicking on the arrow. Each Information we want to find in Excel, such as Author, Year, Title, Journal, Volume (Issue), Pages and Abstract must be added using a new tab We will erase everything on Bibliography by clicking on **Layout**, Close the window and rename the **Style**. In **Endnote** we will click on File and export the references in a text file using the New style created. In **Excel**, we will open the **Text File** and when the windows will be opened, we will click on **Next** until the apparition of **Finish**.
